# An ethical analysis of policy dialogues

**DOI:** 10.1186/s12961-023-00962-2

**Published:** 2023-01-27

**Authors:** Polly Mitchell, Marge Reinap, Kaelan Moat, Tanja Kuchenmüller

**Affiliations:** 1 Centre for Public Policy Research, King’s College London, London, United Kingdom; 2 World Health Organization Regional Office for Europe, Copenhagen, Denmark; 3 McMaster Health Forum, McMaster University, Hamilton, ON Canada; 4 World Health Organization, Geneva, Switzerland; 5 School of Education, Communication & Society, King’s College London, Waterloo Bridge Wing, Franklin-Wilkins Building, Waterloo Road, London, SE1 9NH United Kingdom

**Keywords:** Policy dialogue, Knowledge translation, Evidence-informed policy, Public health, Ethics, Deliberation, Procedural values, Substantive values

## Abstract

**Background:**

A policy dialogue is a tool which promotes evidence-informed policy-making. It involves deliberation about a high-priority issue, informed by a synthesis of the best-available evidence, where potential policy interventions are discussed by stakeholders. We offer an ethical analysis of policy dialogues – an argument about how policy dialogues ought to be conceived and executed – to guide those organizing and participating in policy dialogues. Our analysis focuses on the deliberative dialogues themselves, rather than ethical issues in the broader policy context within which they are situated.

**Methods:**

We conduct a philosophical conceptual analysis of policy dialogues, informed by a formal and an interpretative literature review.

**Results:**

We identify the objectives of policy dialogues, and consider the procedural and substantive values that should govern them. As knowledge translation tools, the chief objective of policy dialogues is to ensure that prospective evidence-informed health policies are appropriate for and likely to support evidence-informed decision-making in a particular context. We identify five core characteristics which serve this objective: policy dialogues are (i) focused on a high-priority issue, (ii) evidence-informed, (iii) deliberative, (iv) participatory and (v) action-oriented. In contrast to dominant ethical frameworks for policy-making, we argue that transparency and accountability are not central procedural values for policy dialogues, as they are liable to inhibit the open deliberation that is necessary for successful policy dialogues. Instead, policy dialogues are legitimate insofar as they pursue the objectives and embody the core characteristics identified above. Finally, we argue that good policy dialogues need to actively consider a range of substantive values other than health benefit and equity.

**Conclusions:**

Policy dialogues should recognize the limits of effectiveness as a guiding value for policy-making, and operate with an expansive conception of successful outcomes. We offer a set of questions to support those organizing and participating in policy dialogues.

## Introduction

A policy dialogue is a tool which promotes evidence-informed policy-making. It involves deliberative discussion, which draws on the best available research evidence, local knowledge and other relevant considerations to identify the best policy solutions to a high-priority public health issue in a particular local context. It typically involves a range of stakeholders – people who are involved in or likely to be affected by decisions taken about the policy issue – and is supported by a facilitator. It is a time-bounded event, usually an in-person meeting which takes place over several hours, or possibly days – though it sits within a broader ‘knowledge translation’ process which may include research, preparation of materials and communication with stakeholders, both before and after the event itself [1]. Policy dialogues contribute, at least in theory, to the development and implementation of good, effective, evidence-informed policies.

Policy dialogues often take as their subject ‘wicked problems’ – issues which resist solution, where there is no clear ‘right’ answer and where a number of different interests, priorities and values are in tension [2–5]. For example, two policy dialogues have been conducted in Moldova considering policies to reduce the consumption and health impact of alcohol – including restriction on alcohol trading, regulation of advertising and taxation-based disincentives – with harmful alcohol use representing a significant health problem in the country. Policy dialogues on the topic of antimicrobial resistance – a global public health priority – have been conducted across the world, including one in Hungary which considered policy options including the development of a national antibiotic stewardship programme and evidence-based clinical guidelines; changes to medical, dental and pharmacy education; and public information campaigns. These issues, like many others in public health, can be difficult to tackle because of conflicting stakeholder interests – including those of government, individuals and commercial actors – and the need for widespread behaviour change to improve health outcomes.

Building on increasing interest in and endorsement of the role of deliberation in evidence-informed policy-making [6–9], ‘policy dialogues’ were promoted under this name by the influential ‘SUPPORT Tools for evidence-informed health Policymaking’ series in *Health Research Policy and Systems* in 2009 [1, 10]. Policy dialogues are part of wider ‘knowledge translation’ efforts in the policy-making domain. Policy-making is notoriously burdened by an ‘evidence-practice’ or ‘knowledge-action’ gap: there can be very good research evidence about the effectiveness (or indeed ineffectiveness) of an intervention in principle, but a variety of obstacles prevent this knowledge being acted upon and put into practice [11]. ‘Knowledge translation’ efforts seek to overcome these impediments by making relevant knowledge that is generated through academic research accessible and useful to policy-makers, practitioners and others, with the objective of improving their decision-making and its outcomes [12]. Policy dialogues encourage stakeholders to bring local knowledge to bear on research findings and can help to secure social and political support for context-appropriate policies, thus providing a means of overcoming key obstacles to knowledge translation and effective evidence-informed policy-making.

While policy dialogues have been used in a wide range of policy areas, in this paper we focus on the public health setting, where this is taken to encompass not just preventative interventions but also other organized measures to promote health and well-being, including health system-level policies and interventions [13]. Policy dialogues are used and endorsed in particular by WHO which has been instrumental in their increased deployment in health policy-making [14–16].

In this paper, we develop an ethical analysis of policy dialogues which can serve to guide those involved in policy dialogues. We largely focus our analysis on the deliberative dialogues themselves, rather than the policy and research context within which they sit; as such we do not discuss important ethical issues such as fair donor funding and good research practices. While policy dialogues involve empirical evidence, they also ‘concern questions which cannot be satisfactorily addressed by purely empirical or logical means’ [17]. They therefore unavoidably require those organizing and participating in them to make judgements about what matters and the relative priority of different ends – issues which cannot be decided by the evidence alone [18, 19]. These judgements will result in different outcomes for real people; sometimes they will be a matter of life or death, or have substantial impact on the health, well-being or life-course of many. Ethical guidance can help decision-makers navigate such ethical dilemmas to make good, well-justified decisions.

There is widespread recognition of the central role of ‘values’ in policy dialogues – both in their design and within deliberations themselves. However, there is little analytical reflection on the values that should guide and shape how policy dialogues are convened and structured, nor on how values should be invoked and examined in the deliberations themselves. Certain guiding procedural values are emphasized in the literature as being reflected in and promoted by policy dialogues, including trust, respect, inclusivity, accountability and transparency, among others [20–25]. But it is typically unclear whether these are necessary conditions for legitimate policy dialogues, characteristics which help policy dialogues achieve their ultimate ends, or valued outputs. Within deliberations, it is widely reported that stakeholders bring their own values – as well as interests, knowledge and understanding of the issues at hand – to the table [1–3, 20, 23, 26–29]. However, relatively little is said about what it would mean for deliberation about values to be better or worse.

In this paper we seek to fill these gaps by developing an ethical analysis of policy dialogues, which aims to help individuals and institutions involved in organizing and participating in them to navigate ethical challenges. Our analysis is structured around three core ethical components of policy dialogues: (i) its goals or ends, (ii) the relevant procedural values and (iii) the relevant substantive values [30]. Justification of decision-making processes typically refers to procedural values, which relate to the decision-making process (how decisions are made), and/or substantive values, which relate to the content (what decisions are made and why) [31]. Procedural values are those features of a decision-making process which lend legitimacy to the process itself, and substantive values are things that it is good to aim at or promote through decision-making. Our ethical analysis goes beyond mere description of the actual goals and stated procedural and substantive values relating to policy dialogues, also exploring what these ought to be.

After briefly outlining our methods in the Methods section, in the What is a policy dialogue? section we focus on the goals of policy dialogues, developing a detailed account of their objectives and characteristics. In the  Procedural values in the policy dialogues and Substantive values in policy dialogues sections we explore, respectively, what makes policy dialogues legitimate and good. By ‘legitimate’ we broadly mean what makes the deliberative process fair or reasonable as a process – in the way that flipping a coin might be a legitimate process for selecting who goes first in a football match, or voting might be a legitimate process for selecting an elected representative. In the Procedural values in policy dialogues section, we argue that transparency and accountability – procedural values frequently invoked to confer legitimacy on policy-making processes – are not central to policy dialogues. Instead, the process is legitimate insofar as it embodies the key characteristics and serves the objectives set out in the What is a policy dialogue? section. By ‘good’ we mean what makes the content of deliberations ethical and why. A decision-making process can be legitimate, but still produce unethical decisions, as when people democratically vote for a cruel or oppressive policy. In the Substantive values in policy dialogues section we argue that good policy dialogues need to actively consider a range of substantive values other than health benefit and equity. In the Conclusions section we conclude and offer a set of guiding questions for those organizing and participating in policy dialogues. Policy dialogues cannot fulfill their core function without a central concern for effectiveness with respect to both policy-making and health outcomes; however, good policy dialogues must recognize the limits of effectiveness as a guiding value for policy-making, and operate with an expansive conception of successful outcomes.

This research was initiated to support the work of the WHO Evidence-Informed Policy Network (EVIPNet), which actively promotes and technically supports the use of policy dialogues by its member countries [32], but the discussion is equally relevant to policy dialogues outside of this context.

## Methods

This paper offers a normative analysis of policy dialogues – an argument about how policy dialogues ought to be conceived and executed. In the empirical bioethics tradition, our research process combined social scientific research methods with philosophical conceptual analysis to generate critical discussion grounded in a realistic representation of the aims and characteristics of policy dialogues [33].

The discussion in the What is a policy dialogue? section is largely informed by a formal literature review. The aim of the review was to identify literature which appraised deliberative dialogue as a policy-making tool, with a view to understanding how ethical values are currently considered in this context. Seven research databases were searched, combining the title search term (‘policy dialogue*’ OR ‘deliberative dialogue*’ OR ‘stakeholder deliberation’ OR ‘deliberative engagement’ OR ‘public deliberation’) with the keyword search term (policy). A detailed search strategy is included in an Annex. The initial search generated 283 results. After removing 86 duplicates and four book reviews/conference abstracts, the titles and abstracts of the remaining 193 results were reviewed to exclude those concerning non-dialogic stakeholder engagement and public engagement, and those that did not appraise policy dialogues or deliberative dialogues as a method. Papers which took a specific policy dialogue as their subject were not excluded if they also offered some degree of analysis of the method itself. Of the 61 results designated for full text review, one paper was inaccessible. A further five papers were excluded after full-text review. Thematic analysis of the remaining 55 papers generated the objectives and characteristics of policy dialogues as detailed in the What is a policy dialogue? section, and also identified explicit invocation of values or ethics in the literature.

The discussion in the Procedural values in policy dialogues, Substantive values in policy dialogues and Conclusions sections is informed by an interpretative literature review. Relevant literature was identified using authors’ existing knowledge of public health ethics and deliberation, by hand searching reference lists and on the recommendation of colleagues. This included grey literature – such as WHO and EVIPNet literature on policy dialogues and evidence-informed policy-making, evidence briefs and policy dialogue summaries from past EVIPNet policy dialogues – as well as theoretical and ethical analysis of public health, public policy decision-making and deliberation. We endeavoured to augment and interpret the findings from the formal review and to subject the literature on policy dialogues to critical reflection – including reflection on concepts and issues that are absent or overlooked. Both the formal and interpretive aspects of this research were designed to generate a rich and realistic understanding of policy dialogues and the context in which they operate, rather than an exhaustive appraisal of the approach or the field.

## What is a policy dialogue?

There is some variety in the way that policy dialogues are defined and understood, so this section scopes out the territory. First, we outline what a policy dialogue is for, what its objectives are and how it fits into the policy-making process. Second, we identify the core characteristics of policy dialogues. In practice there is variation in the aims and configuration of different policy dialogues. Such variety does not always indicate poorly conceived and executed policy dialogues; being attuned and responsive to its context is essential to a policy dialogue successfully contributing to better policy-making. However, based on our review of the published literature on policy dialogues, a cluster of shared features and aims can be picked out.

### Objectives of policy dialogues

As knowledge translation tools, one of the objectives of policy dialogues is to generate, or contribute to, policies that are informed by research evidence. But health policies being ‘evidence-informed’ is chiefly of value insofar as it is instrumental to them achieving desired or good outcomes. It is not beneficial to have policies that are informed by evidence but do not result in improvements in population health and well-being or the functioning of healthcare institutions. An intervention may be efficacious in principle – it has worked under some conditions – but that is no guarantee that it will produce the same effects in other contexts. The ‘same’ intervention may have very different effects when implemented in an environment with different political, economic, cultural and social institutions, resources and conventions. So a principal objective of policy dialogues as knowledge translation tools is to ensure that prospective evidence-informed health policies are appropriate for and likely to be effective in a particular context.

Policy dialogues aim to contribute to context-appropriate policy-making in – very broadly – two ways. The first is to identify policy options that are likely to work in the context. This will involve consideration of research evidence, knowledge of local systems, institutions and attitudes, but also consideration of political will – whether decision-makers are likely to be motivated to support and enact the proposed policy. But, secondly, policy dialogues also have the potential to facilitate the success of policies more directly, by creating conditions in which a policy is more likely to be effectively implemented. A good policy dialogue will involve at least some of the people on whose action and agreement successful policy-making depends. By establishing relationships between key actors and institutions, building trust, generating alignment, shared understanding and shared commitments, and motivating action, policy dialogues can change the policy-making landscape to be better at developing and implementing policies [29, 34].

This discussion indicates that policy dialogues sit between, and aim to mediate between, research and politics. As knowledge translation tools, they are premised on the recognition that evidence is not the only factor that shapes policy decision-making. The aim of policy dialogues is not to preclude other factors – such as political expediency, interest-group pressure, electoral incentives and institutional constraints – from playing a role in policy-making, but rather to ensure that research evidence plays a decisive role in policy decision-making alongside other factors. They thus seek to position evidence as a node around which alignment and policy action is built. A normative commitment to the value of evidence in policy-making thus underpins and drives policy dialogues.

### Characteristics of policy dialogues

We highlight five core features of policy dialogues. Policy dialogues are (i) focused on a high-priority issue, (ii) evidence-informed, (iii) deliberative, (iv) participatory and (v) action-oriented. Other core features have been suggested, including use of a facilitator [35], following a rule about not attributing comments to individuals and not aiming for formal consensus [1], as well as provision of adequate time and resources [22, 36, 37]. While such factors support the successful attainment of the five core characteristics, we consider them secondary characteristics. Without one or more of the core features, it is dubious whether an event is a policy dialogue at all; without one of the secondary characteristics, it is merely liable to be a bad policy dialogue. The five core characteristics described here are inter-defined and not easily separable.

#### (i) Focused on a high-priority issue

Policy dialogues take as their subject a high-priority issue. Ideally this is a topic that is considered a high priority for policy action by all stakeholders, but this will not always be achievable [1]. It is vital, however, for the topic to be recognized as a priority by at least some actors with genuine decision-making power, ideally who are involved in the policy dialogue – for if it is not, then it is difficult to see how it can motivate responsive policy action. Wider recognition of a policy area as a health priority may also be necessary for the success of policies, particularly when the issues they address have proved difficult to resolve [3].

While the topic of a policy dialogue is determined in advance, discussion about the appropriateness of the topic and the way issues are characterized and framed can be part of the policy dialogue itself [38, 39]. Some disagreements can be resolved with reference to evidence or expert opinion [25], but sometimes there will be disagreement about the validity or appropriate source of evidence [21]. To avoid such disagreements entirely scuppering the policy dialogue process, there will often be considerable dialogue and stakeholder engagement prior to the policy dialogue itself – to ‘sense check’ the topic, its characterization and the suggested policy options to be shared with participants [29, 40].

#### (ii) Evidence-informed

Policy dialogues are informed by evidence. Typically, participants will receive a pre-circulated evidence brief introducing the best-available evidence about a problem, its causes, the most promising policy solutions and identifying potential barriers to implementation [1]. This provides some common ground and a starting point for discussions, though the findings and framing are open to critical examination [41]. Evidence will be synthesized and briefs prepared by a research team, who may work in collaboration with other policy actors or stakeholders. Value judgements unavoidably play a role here – in determining what evidence to draw on and prioritize, for instance, and in evaluating the plausibility of policy options. But the invocation of evidence should aim to provide a rounded and relatively impartial picture of the terrain, to ensure that deliberation is not narrowly constrained from the outset [42].

Many kinds of research evidence can be used to prepare an evidence brief, including findings from controlled trials, observational studies, systematic reviews and meta-analyses, indicating the effectiveness of public health interventions; local observational and administrative data describing the magnitude of the problem; qualitative studies portraying views about and experience of the problem and potential interventions; local cost-effectiveness studies; and process evaluations explaining how and why interventions worked in a given context. But policy dialogues also involve forms of evidence which may not be captured in formal research processes: ‘tacit knowledge’ captures the knowledge, skills and understanding gained through experience that is difficult to codify [1, 43–45]. It may include understanding of policy-making institutions and the health system, cultural attitudes and conventions, and the historical success of policies and policy decision-making. Policy dialogues aim to contribute to the development and implementation of policies that are in fact effective and appropriate. This requires moving beyond idealized representations of the functioning of government and policy-making institutions and the capabilities of health systems and being realistic about how institutions and systems actually work in practice. Identifying relevant tacit knowledge will depend on participants having had a particular set of experiences, so involving the right mix of stakeholders is crucial [23, 44, 46, 47].

#### (iii) Deliberative

Policy dialogues are deliberative – indeed they are sometimes known as ‘deliberative dialogues’[2, 14, 26, 41, 48]. Deliberation is a form of, or a part of, decision-making which is chiefly characterized by reflective consideration and discussion of advantages and disadvantages of different options [7]. Giving opinions or voting on the best solution to a problem does not amount to deliberation; rather, deliberation involves identifying the full range of reasons for and against different options and reflecting on the relative balance of reasons [38, 49, 50]. Deliberation should begin with genuine openness about the alternatives under consideration – there should not be a prior presumption of correctness of any option. Deliberation is an aid to thought and judgement, but a decision is not prescribed by deliberation, as might be the case with more algorithmic or rule-based decision-making [7]. This means that with a different group of people or at another time different decisions might result.

In contrast to debate – which is typically oppositional and where participants look for weaknesses in their opponents’ argument – deliberative dialogue is constructive and collaborative and seeks common ground and agreement [1]. Deliberative dialogue is a valuable tool for addressing intractable problems because it encourages critically examining a problem, trying to re-frame it from different perspectives, and considering trade-offs that are involved in different ways of conceptualizing and managing it. In the context of policy dialogues, deliberation is pragmatic, aimed at finding solutions and motivating action, rather than just constructing logical arguments.

#### (iv) Participatory

Policy dialogues are participatory. They include stakeholders who are involved in or affected by a policy decision – individuals or institutions whose knowledge, action or acquiescence is necessary for an effective public health policy to be developed and implemented [1]. Typically, stakeholders will include representatives of policy-making institutions, such as the ministry of health, other government departments or public health institutions, as well as academics and practitioners in fields related to the topic in question. Sometimes it may be necessary to include representatives of commercial organizations, as public health issues often relate to commercial products such as high in fat, salt and sugar (HFSS) food, sugar sweetened beverages, alcohol and tobacco. Stakeholders may have substantially diverging beliefs, values and motivations relating to the public policy issues under consideration.

As stakeholders are selected on a largely pragmatic basis, on account of their capacity to contribute to policy change, some of the considerations of fair and representative selection of participants that might be relevant to other deliberative and consultative policy-making processes do not apply. Many participants will have a conflict of interests, insofar as they and their institutions will be substantially invested in and affected by any policy change. However, sometimes very strong conflicts of interest may make it prudent to exclude stakeholders from discussions on pragmatic grounds; for example if strong commercial lobbying has been a barrier to policy change in the past, commercial representatives may not be included. Patient or public representation will not be part of every policy dialogue – policy dialogues are not primarily public engagement processes. When public participation is not necessary for the successful development and implementation of health policies, inviting members of the public to policy dialogues may be counterproductive, for example insofar as it hinders open discussion of barriers to policy implementation. Members of the media are typically not invited. Policy dialogues operate under the Chatham House rule, which means that any subsequent discussion of their content must not attribute any comments or views to an identifiable individual [26, 29].

There is a balance to strike between including representatives of all relevant groups and institutions and including a small enough number of people that deliberative dialogue remains possible [35, 44]. Moreover, merely inviting the right people is not sufficient for a policy dialogue to be considered participatory – participants must also meaningfully contribute to deliberations [45, 51]. A good facilitator will ensure all participants have an opportunity to contribute, including those with minority views, people from marginalized groups, and less powerful social actors [16]. Including relevant stakeholders in policy dialogues is instrumental to the success of policy dialogues in identifying effective and appropriate policy solutions, and being realistic about their implementation barriers [16, 28]. Inclusion can also play a legitimizing function – in relation to some social issues and policy decisions, the acceptability of decision-making further down the line depends on the meaningful inclusion of relevant stakeholders earlier in the process [25, 37, 42].

#### (v) Action-oriented

Policy dialogues are action-oriented. What this means in practice is somewhat nuanced. Policy dialogues aim to generate successful policy change and improved health outcomes, but they do not typically have formal decision-making power. Participants in policy dialogues will be employed or engaged by institutions with their own decision-making processes and practices which cannot be bypassed. Even if participants are aligned on the best policy solution to a given problem, the details of the policy still need to be specified and individuals need to consult with their respective institutions or garner wider agreement before policy change can be effected [14]. Policy dialogues are designed to disrupt the normal policy-making process by changing the way that stakeholders communicate and think about a policy issue, but they do so largely within existing institutional processes.

Policy dialogues do, nonetheless, try to build towards common action [23]. They achieve this chiefly by seeking agreement among key stakeholders about how to characterize the problem in question and what the most promising policy solutions are, and ultimately generating motivation and shared commitments in actors who have decision-making power and policy influence [23, 24, 44]. Deliberation is the tool used to secure such alignment. While this process is sometimes described as building consensus [23, 24, 29], formal consensus on all questions is not required and a policy dialogue is not a failure if differences of opinion between stakeholders remain at the end [1, 39, 43, 52]. Policy dialogues do not typically involve voting or other aggregative decision-making procedures to generate a single decision outcome. Rather, policy dialogues aim to build trust and mutual support between participants and to secure agreement on certain core issues, with the intention that stakeholders will continue to work, separately and together, in their respective institutions and domains, in pursuit of shared commitments [23, 24, 47, 53]. A majoritarian decision procedure may work against this by marginalizing or silencing participants in the minority, and risks undermining the prospect of change in policy and practice. Nonetheless, points of agreement may be amplified and embraced if they do arise during deliberations.

Policy dialogues can give stakeholders tools – evidence, arguments, motivation and support from others – for making changes within their respective domains and institutions. But the positive changes generated by policy dialogues need not be limited to the development and implementation of discrete policies. Policy dialogues can successfully contribute to better policy-making not only by having their arguments and recommendations taken up by decision-makers, but also in less direct ways – by influencing the way that a topic is framed and characterized in public discourse; by raising the profile of an issue and promoting public and institutional awareness; by making it difficult to ignore existing evidence in future decision-making.

One potential weakness with this softer sense of ‘action-oriented’ is that it can generate apparent but not actual buy-in from stakeholders. Participants can agree or seem to agree on the day but not follow through on commitments afterwards [15], or genuine intention to act by stakeholders who participate in policy dialogues can be knocked back by senior decision-makers once they take ideas and recommendations back to their institutional setting [23]. This can be managed and mitigated, at least to some extent, with ongoing communication and support after the policy dialogue itself. But this risk is to some extent necessary; policy dialogues largely operate within existing policy-making institutions and processes, rather than seeking to supersede them. The use of ‘soft power’ rather than coercive or mandated authority is part of a broader strategy to generate meaningful and sustainable change through genuine stakeholder buy-in and alignment.

#### Core characteristics in practice

In practice, events which are called policy dialogues often fail to embody the objectives and characteristics set out in this section. They may use poorly constructed evidence briefs, exclude relevant stakeholders, be stymied by asymmetrical power relations, include inadequate interrogation of the relevance of claims made by participants or remain characterized by intractable disagreement [27, 40, 54, 55]. For these reasons, or others, they may not in fact contribute to the development and implementation of evidence-informed and effective policies, and so fall short of fulfilling their knowledge translation function. We assume throughout this paper that policy dialogues can, and at least sometimes do, achieve their objectives, enabled by their core characteristics. This is not unfounded. Policy dialogues have, in practice, been part of successful public health policy-making since the late 2000s. The 2017 policy dialogue on alcohol control in Moldova was used to secure parliamentary support, which led to legislative changes including recognizing beer as an alcoholic beverage, restrictions on the advertisement of alcoholic products and increased taxation – topics and policy options which had been discussed in the policy dialogue. In Canada, a stakeholder dialogue convened in 2017 to discuss the creation of a National Pain Strategy resulted in agreement around the need for a national coordinating body [56]. This alignment was leveraged to help establish the Canadian Pain Task Force, which led to the development of an action plan for pain in Canada.

Despite promising examples, there is limited evidence about the specific effectiveness and contribution of policy dialogues to policy-making and health outcomes – in part because designing research to meaningfully measure this poses a number of challenges [57]. Rarely will there be a comparative ‘control’ scenario against which to measure the impact of policy dialogues. The impact may include subtle but distinctive changes in the way that institutions or decision-makers frame or address an issue. And successful policy-making depends on a range of factors outside of the control and feasible scope of policy dialogues. This does not mean that policy dialogues cannot fulfill their objectives, but it does suggest it will be difficult to determine with certainty exactly how and when they do so.

The five core characteristics identified here are not simple, formal attributes; each represents a complex, normative, aspirational quality. Nor are they best understood as a checklist of key criteria. The characteristics of policy dialogues discussed above need to be understood in relation to one another: what it means for a policy dialogue to be ‘participatory’, for example, is shaped and constrained by what it means for it to be ‘deliberative’ and ‘action-oriented’. The characteristics therefore need to be balanced and mutually negotiated – in some ways they will be complementary and in other ways they will be in tension. The appropriate balance of characteristics needs to be understood in relation to the broader objectives of policy dialogues – whether the process is in fact liable to promote and generate good public health policies and outcomes in this context. This knowledge translation function should be understood relatively broadly and not just in relation to discrete policy outputs. But unless a policy dialogue is oriented towards its knowledge translation objective, it is difficult to understand how it can be characterized as policy dialogue at all, let alone a legitimate or good policy dialogue.

## Procedural values in policy dialogues

Procedural values are features of a decision-making process which lend legitimacy to the process itself or its outcomes [31]. In an environment of reasonable disagreement – where different people have reasonably different views on what matters and the relative value of different ends – the use of a fair procedure can be invoked as a way of securing justification for decisions in the absence of substantive moral agreement [58]. In the context of policy dialogues, this would imply that convening and setting up a policy dialogue in the right way can ensure, or at least greatly increase the likelihood, that the decisions and actions which it generates are justified. This is ostensibly plausible. However, in this section, we argue that use of a legitimate process is not enough to secure good policy dialogues nor good outcomes – policy dialogues must be judged, at least in part, by the substantive content of deliberations and their impact.

Perhaps the most notable ethical framework for health policy decision-making is the Accountability for Reasonableness (A4R) framework, spearheaded by Norman Daniels and James Sabin [58, 59]. The A4R framework sets out four procedural principles to govern decision-making about healthcare priorities. Decisions which meet these conditions can be said to be fair and legitimate. Their ‘publicity’ condition requires decisions and their underlying rationales to be made public. A ‘relevance’ condition demands that the rationales in question are ‘reasonable’, that is, they appeal to reasons that will be accepted as relevant by fair-minded people who want to find mutually justifiable terms of cooperation. A ‘revision and appeals’ condition requires that there are mechanisms for challenging and revising decisions in light of new evidence or arguments. And, finally, a ‘regulative’ condition requires regulation of the process to ensure these three conditions are met. These four conditions together seek to make the process accountable, by requiring decision-makers to give clear, comprehensible and acceptable reasons for their decisions. The A4R approach is very widely lauded and invoked, especially in relation to health technology appraisal (HTA) processes, which assess the cost-effectiveness and appropriateness of novel therapies and technologies [49, 60], but also in relation to priority setting and policy-making decisions more broadly [61, 62].

It is not difficult to see how A4R might be extended to cover policy dialogues. While policy dialogues go beyond priority setting in their scope, they also concern decisions that rest on value judgements about which reasonable people disagree. Indeed, the need for policy dialogues arises in part because there is deep and intractable disagreement about the best way to understand and resolve public health issues. A4R seeks to sidestep at least some of this disagreement by providing a structure for decision-making which legitimizes outcomes without needing to resolve all ethical disputes. It permits decision-makers to settle on a reasonable solution without demanding that it is the best of all solutions. In this sense, it is relatively pragmatic and action-oriented – preferring an achievable ‘good enough’ solution to an ideal, and potentially unachievable, ‘best’ solution. Despite appearances, A4R is not a suitable ethical framework for policy dialogues, because transparency, accountability and the relevance condition have limited applicability as guiding procedural values.

### Counterproductive transparency

Transparency is increasingly taken to be a non-negotiable component of public decision-making [63]. Scrutiny of public processes, of decisions and of underlying evidence and reasoning is seen as necessary to ensure that public institutions genuinely act and operate in the public interest, and to establish a basis for citizens to trust their representatives [64, 65]. While transparency is sometimes treated as an unqualified value, it is important to remember that within political institutions and the policy-making process, transparency is instrumentally valuable [66]. It is possible to ask whether mechanisms of transparency in fact secure better, less corrupt, less partisan, more acceptable decision-making – if they do not then their value is, to that extent, limited. There may then be aspects of public decision-making which are not best served by transparency [67].

The content of policy dialogues is not, typically, available for public scrutiny. While a short summary may be produced and published, this need not contain much detail about the content of deliberations. The use of the Chatham House rule means that, while participants can voluntarily identify their own views to their colleagues or others, any other views must remain anonymous. Participants in policy dialogues are expected to take any conclusions and recommendations back to their respective domains of influence and to try to act on them or urge action to be taken on them, so policy dialogues should not be understood as entirely closed and private affairs. But nor are they best described as ‘transparent’ where this means that they are made available for public scrutiny. We suggest that this is justified: making policy dialogues transparent risks seriously undermining their capacity to contribute meaningfully to better policy-making.

Policy dialogues occur at a formative stage of the policy-making process. They offer an opportunity to try out ideas, to explore unconventional options freely and to experiment with different ways of framing problems [2, 23]. Opening such tentative deliberations and conversations up to public scrutiny risks changing their nature – if participants are concerned about saying the ‘wrong’ thing, revealing their ignorance, being held to account for something they casually suggest, or being criticized or humiliated for their opinions, they will be much more guarded about what they say [66]. This is one of the key reasons that media representatives are not typically invited to policy dialogues. Policy dialogues are intended to disrupt policy-making by creating a space where stakeholders can, temporarily, step away from and challenge some of the assumptions and conventions of their institutions [3, 23, 29]. Constructing new, shared understanding of issues and motivation to act on them depends on the possibility of genuine openness to new ideas and options. But it is difficult to see how this can be realized if participants are incentivized to reproduce, rather than challenge and break down, the official, public views of their respective institutions. Moreover, policy dialogues require genuine openness about institutional motivations, obstacles to policy adoption and implementation, and the political and social reality in a given context [29, 41]. This is essential to their fulfilling a knowledge translation function – without such openness, many of the existing obstacles to effective policy-making remain firmly in place. But a transparent, public process will strongly discourage openness. The non-attribution of comments is deemed essential to generating a non-confrontational, productive atmosphere [23].

Transparency can then be in tension with the aims of policy dialogues. This is not to say that transparency is never valuable as part of policy dialogues – making an issue public may sometimes be a good way of motivating action on it. The fact that a policy dialogue is being held is unlikely to be kept secret, and often certain key documents, such as a policy brief and a brief summary of a dialogue, which does not attribute comments to specific individuals, will be published to support ongoing decision-making. Nonetheless, transparency is not a prominent procedural value for policy dialogues and what value it has will operate chiefly in service of other objectives.

### The limits of accountability

Somewhat like transparency, accountability is ‘one of those golden concepts that no one can be against’ [68]. In the public sphere, it is widely invoked as a means of increasing the effectiveness and efficiency of public institutions. But, like transparency, the relevance of accountability to policy dialogues is limited. While accountability is sometimes assumed to be exhausted by transparency, it is best understood to be broader in scope [66]. Accountability requires the accountable party to both inform about and provide justification for their actions, but also involves an accounting party with the capacity to pass judgements and impose sanctions or other consequences [68, 69]. Unlike HTAs, policy dialogues are not strictly decision-making mechanisms. It is therefore not clear how sanctions might apply to policy dialogue participants if they fail to act appropriately, nor what holding policy dialogue participants responsible for the failure of a policy might involve. Without retracting the Chatham House rule, it is not possible to hold individuals responsible and, as stakeholders of different organizations and groups, policy dialogue participants do not really constitute a corporate agent which can be sanctioned or suffer consequences. It is likely to be difficult to attribute the failure of a policy to a policy dialogue per se – the success of policies will also depend on subsequent decision-making about the design, adoption and implementation of policies, as well as features of the environment and population in which it is implemented. Because policy dialogues are not formal decision-making mechanisms, and not amendable to traditional accountability mechanisms, A4R’s ‘revision and appeals’ condition is also limited in its applicability to policy dialogues.

Policy dialogues might, however, be thought to contribute to and promote accountability in policy-making processes more broadly. If accountability involves not just informing the accounting party about actions and decisions, but also explaining or justifying them, then policy dialogues potentially play an important role in such accounts. For, if they go well, policy dialogues produce rich and reflective rationales for their conclusions and policy recommendations – both in relation to ideas and policy solutions that they favour and those that they ultimately reject. Whether decision-makers participate in policy dialogues themselves or whether they receive recommendations and suggestions through subsequent communications, these rationales will be central to justifying policy decisions based on policy dialogue deliberation. It may, to a certain extent, also promote transparency in policy-making, if rationales are shared publicly. However, this does not mean that accountability is a critical procedural value for policy dialogues themselves – more important is that they involve meaningful, reason-giving deliberation, to enable them to play a role in wider accountability structures in policy-making.

### Relevance through deliberation

The relevance condition of A4R is ostensibly more promising as a procedural value for policy dialogues. Good policy dialogues aim to construct shared rationales and arguments, which will be acceptable not just to participants but to their colleagues, policy decision-makers and the wider public. This chimes well with the idea that they should appeal to reasons that will be accepted as relevant by people who want to find mutually justifiable terms of cooperation. This does not mean that rationales and decisions have to please everyone, but it does mean that reasons need to be broadly acceptable to those who want to see action taken to address a given issue.

Daniels and Sabin invoke a procedural approach to avoid having to settle substantive ethical disagreement. However, they understand fair process to be a practical necessity rather than something that is intrinsically morally required for good decision-making [70]. A4R facilitates decision-making and circumvents intractable disagreement by tolerating ‘good enough’ justifications and decisions, rather than demanding the best solution be found. Policy dialogues can be seen as offering an alternative solution to the same problem – rather than trying to circumvent the disagreement using formal procedures, they apply a deliberative lens to disagreement to try to work out the best solutions to complex practical problems. Seen in this light, it is easier to understand why a procedural framework like A4R is not particularly helpful for understanding policy dialogues. Rather than seeing relevance as a kind of formal constraint on reasons which precedes decision-making, as A4R portrays it, policy dialogues see relevant reasons as something that is created through deliberation on substantive values. This means that, to understand whether policy dialogues are legitimate, we need to look at the substantive content of deliberations.

This helps to clarify the status of procedural values in policy dialogues more generally. As knowledge translation tools, policy dialogues exist to support and contribute to evidence-informed policy-making and, ideally, better health outcomes. The value of policy dialogues as a policy-making procedure is largely instrumental in relation to this end. This is not to say that a well planned and executed policy dialogue which fails to lead to policy change because of bad luck is thereby illegitimate – for example, if an economic or environmental crisis or a health emergency radically changes government priorities in the aftermath of a policy dialogue. Moreover, getting a particular policy enacted or securing a particular health outcome is not the only measure of a successful policy dialogue – its impact on the policy-making process can be more subtle, involving increased awareness of a topic, changes to the way an issue is framed and alignment of key policy-makers, and increased understanding of the evidence base.

In general, as long as effectiveness is understood expansively, and not just in relation to narrowly defined ends, the things that legitimize policy dialogues are the things that make them effective as policy-making and public health tools. In the What is a policy dialogue? section we outlined five core characteristics which help policy dialogues to achieve their objective as knowledge translation tools. We therefore suggest that these characteristics can be seen as guiding procedural values for policy dialogues. As we indicated in the What is a policy dialogue? section, these characteristics are normative, aspirational and goal-directed, and need to be understood in relation to the effectiveness of policy dialogues as policy-making tools. All these characteristics also require the genuine engagement, openness, commitment and motivation of participants – they cannot be imposed and they are not formal rules or checks which can be applied to the process. Importantly, this cluster of features create the conditions for an effective policy dialogue but they do not secure or entail it. The effectiveness, and so the legitimacy, of a policy dialogue will depend not just on formal features of the process, but on the substantive content of deliberations – the specific reflection on and decisions about the issue and policy options under consideration – and the consequences and outcomes that deliberation generates.

## Substantive values in policy dialogues

In this section we consider substantive values in policy dialogue deliberations. We explore, first, which values should be invoked in policy dialogue deliberations and, secondly, what identifying and ‘balancing’ or ‘trading off’ values involves in practice.

### Beyond population health

The literature on policy dialogues is, on the whole, fairly silent about what good deliberation about values looks like and whether there are any constraints on the invocation or consideration of values. Values are presented as something that different participants bring with them to policy dialogues, along with their experiences, knowledge, interests and preferences [1, 20, 23, 27, 29]. They are part of what will be negotiated and discussed in deliberations [29], and may represent points of tension and disagreement [2, 23], though policy dialogues ideally drive towards identifying or constructing shared values [20, 24, 26]. Values, in this sense, are essentially seen as different people’s sense of what matters, and they are not neatly distinguishable from interests, personal commitments and preferences. There are some values, however, which are largely set outside of this personal framing. The literature on policy dialogues places a near unanimous emphasis on population health – a concern with interventions that improve health outcomes sits at the centre of a policy dialogue’s consideration of any public health issue. This is accompanied by a concern with equity – though this is typically presented as a secondary consideration [1, 25, 40, 47, 71, 72]. That is, it is essential to consider what the impact of different policies will be on different population subgroups, particularly those that are marginalized or vulnerable, and how policies will affect existing health inequalities. These two values, above others, are positioned as non-negotiable components of policy dialogue discussion and decision-making, and are central to determining which policy options are suggested to policy dialogue participants in evidence briefs [73].

This concern with population health and equity, above other considerations, is not surprising, nor does it appear misguided given the broader aims of public health. Public health, as a field of practice, is concerned with protecting and promoting the health of a community [74]. It takes a population perspective, developing and promoting interventions which impact on the health outcomes of large numbers of people within a given population or population subgroup – not just at the individual level [75–78]. The focus of policy dialogues on health benefit reflects this emphasis in public health more broadly. Reducing health inequalities is also an important goal of public health [75, 79]. Looking at population health and welfare in the aggregate can be of limited value in describing the health of a population if distribution of these goods is uneven. Understanding differences in the health of population subgroups provides a richer and more detailed account of population health than just knowing aggregate health benefit. If an intervention delivers health benefits to only some members of a population, and particularly if it widens existing health inequalities, the sense in which it protects and promotes population health is limited. Equity is also seen as valuable in its own right, and its pursuit can be justified even when it involves missing out on opportunities to increase total health [80]. But the emphasis on equity in policy dialogues also makes sense in relation to a public health focus on population health outcomes.

Notably absent from most of the literature on policy dialogues is autonomy, a value which is typically presented alongside health benefit and equality in public health ethics frameworks [75, 79–81]. Other values that are not emphasized in the literature on policy dialogues, but which are plausibly relevant to public health, include privacy, confidentiality, trust (patients’ trust in clinicians, healthcare professionals’ trust in administration and leaders, and citizens’ trust in government for example), solidarity, dignity and professional discretion. Public health policies can positively or negatively impact people and societies in relation to these ends. Public health policies that prevent people from buying alcohol or tobacco at certain times, or which place age limits on their purchase, for example, restrict the autonomy of some or all citizens. Policies that track people’s whereabouts, in the context of infectious disease outbreaks, for example, infringe on their privacy. Even if the costs they involve are justified given the benefits they generate, it is in principle relevant to deliberation and decision-making about such policies to consider their impact in relation to a wider range of ends than health benefit and equity impact.

One way in which such values may be relevant is if a policy option negatively impacting autonomy, privacy, dignity or another valuable end is likely to undermine its ability to generate health benefit, for example by making it unacceptable to people. On this framing, what makes the policy in principle a ‘good’ public health policy is ultimately its impact on population health. However, other values are relevant insofar as they affect the implementation of a policy which is likely to be effective in generating health benefit. But there remains a further question as to whether other such values should be considered, in policy dialogue deliberation and in the evidence briefs which inform discussion, as ends in themselves. Treating other values as ends in themselves might involve considering their relevance to public health decision-making regardless of whether they are liable to make policy options unacceptable or not. It might also involve seeing them as costs or benefits of a policy alongside its impact on population health, rather than just as implementation considerations.

While generating health benefit is central to public health endeavours and will inevitably be dominant in any discussion of issues and policies in this domain, we suggest that there is reason to operate with a broader conception of ‘good’ outcomes in policy dialogues. The policy-making agenda has already broadened to include equity as a discrete end, but it should open up further still and recognize a number of other social goods. Policies which concern the provision of healthcare services may, in particular, require a shift in priority away from population health and towards individual values, such as autonomy and privacy.

Too great an emphasis on health benefit conceptualizes the success of policies in relatively narrow terms. A policy, intervention or method can be effective at generating health benefit while also generating other problematic effects – it can achieve its intended ends inefficiently, generate harmful ‘side effects’ or ‘collateral damage’, or change or contribute to changed attitudes and culture in particular institutions or in society in negative ways, triggering anxiety, mistrust or panic. Policies and approaches that effectively generate health benefit can also have effects that are not unwanted or unintended but are nonetheless ‘costs’ or ‘burdens’ in some sense. Sometimes the success of policies as designed is conditional on individuals bearing certain burdens – for example, by making it more difficult or impossible for them to make certain choices that they otherwise would make. These costs of public health policies might not detract from their expected health benefits, narrowly defined, but they can more clearly be identified as costs when taking a broader view of people’s lives. Kass suggests these costs fall into three main groups: risks to privacy and confidentiality; risks to liberty and self-determination; and risks to justice [82]. We would add relational risks to this list – as health policies and practices have the potential to change the way that people relate to one another, whether that is relationships between patients and clinicians, between clinicians and healthcare leaders, between citizens or residents and government, or just between individuals [83]. Any contribution of health policies to a climate of mistrust, suspicion, animosity or social division should also be seen as a cost.

Thinking about what is good for people more broadly, including consideration of their preferences, is consonant with the expansive conception of health that is characteristic of public health approaches, perhaps best epitomized by the WHO’s definition of health as: ‘A state of complete physical, mental and social well-being and not merely the absence of disease or infirmity’ [84]. Public health is concerned with people’s lives taken in their entirety, not just narrowly defined health outcomes [85]. This broader concern is, in large part, due to the close causal relationship between clinical health outcomes and psychological well-being and social determinants of health [86]. Contextualizing health and health outcomes in a broader social context highlights the relationship between more narrowly health-related ends and other values and social ends. This suggests that consideration of values other than health benefit and equity in policy dialogues is not just necessary for pragmatic ends, to secure motivation and policy action, but also necessary for achieving good health policy and good outcomes in this broader sense. Identifying something as a high-priority public health issue can involve a recognition that it is necessary to prioritize tackling it and to focus on population health over other personal and social goods and projects – that is just what ‘high priority’ means. But this does not mean that other personal and social ends can be abandoned entirely. An issue being high priority does not imply that it is the only priority, or that it should be pursued at all costs.

We have suggested that policy dialogue deliberations should develop and operate with a broad conception of the impact and outcomes of policy decision-making. But this has the potential to introduce more ethical problems than it solves – for other personal and social ends can be, and often are, in tension with population health and equity. In practice there are likely to be trade-offs required between health ends and other values – just as trade-offs are necessary between total health benefit and equitable distribution of health benefit. If understanding what makes policy dialogues good requires attention to their substantive content, then it is essential to consider how the list of relevant values should be decided and what ‘balancing’ or ‘trading off’ values should involve in practice.

### Deliberating about values in practice

In this section we say more about, first, how to approach identifying relevant values for policy dialogue deliberations and, second, how to think about trading values off against one another.

There is no rulebook of values, no external standard to tell us definitively what matters and how much it matters. All we have is the things that in fact matter to us. While there is a philosophical tradition which tries to secure objective, universal values, this approach is at best inappropriate for policy dialogues. Even in the face of widespread agreement about what matters – it is relatively undisputed that happiness, equality, fairness, freedom, respect and kindness are valuable ends (to name but a few) – the practical implications of this remain unresolved. Agreement that something is an important value does not entail agreement that public institutions should promote or try to secure it, for example. And agreement about the importance of some abstract end does not entail agreement about how to define it, how to know whether and when it has been achieved, and its importance relative to other priorities. Given that policy dialogues aim to discuss – and to work towards action on – concrete, context-specific health policy issues and decisions, insisting on a list of pre-determined values, or an approved value framework, prior to deliberation is a strategy for failure. On the other hand, this need not mean than anything goes. A value does not become irrelevant for a policy question just because someone says that it does not matter and it does not become relevant just because someone insists that it is. To some extent, this will be settled by the topic under discussion – autonomy is unlikely to be central to deliberations unless a restrictive policy is imposed and professional discretion will come up for discussion only to the extent that policy options impact on clinical or professional practice, for example. But in less clear-cut cases, the relevance and meaning of values must be subject to deliberation. Deliberation is not the only possible way of identifying and defining values – other approaches might involve mass survey, theoretical moral reasoning, or direct top-down decision-making – but it is the main tool that is used by policy dialogues.

In practice, identifying relevant values is rather more straightforward than it might seem in the abstract. Rather than asking policy dialogue participants to reflect directly on the ethically relevant criteria relating to introducing this policy, a less abstract, and potentially less intimidating, starting point might be reflection on the concrete impact and consequences of the policy option: Who will be impacted by the policy and in what ways? How will it make them feel? What costs and benefits are not captured in the evidence? What costs and benefits are difficult or impossible to measure? What might the broader societal or institutional consequences of this policy be? How might things go wrong? These are ways of asking people what matters, which stay connected to the concrete characteristics of the issues under discussion but which encourage critical reflection on the broader impact of policy-making. This should not be seen as consideration of whether an otherwise good policy is realistically implementable or not but a matter of whether it is a good policy or not. These are not straightforward questions and there may be disagreement about the answers, but part of the role of deliberation is to elicit the tensions and disagreement that characterize decision-making about complex social issues, rather than to mask it.

One concern here might be that this approach is not sufficiently ‘evidence-based’. It requires participants in policy dialogues to pass judgement on the likely effects and impact of policies in absence of research evidence. But personal experience as a source of evidence is already central to policy dialogues – invoking the appropriate tacit knowledge for good decision-making depends on participants having certain personal experiences of institutions, processes and cultural contexts. Expert judgement and critical reflection on evidence is already built into the policy dialogue process and its value is recognized. Ethical reflection on the broader impact and implications of health policies can be understood as an extension of this. As well reflecting on their experience as professionals, participants can also valuably reflect from their perspective as citizens, as parents and children, as patients and users of health systems, as humans. Asking policy dialogue participants to reflect personally on policy decisions highlights the importance of policy dialogues disrupting institutional decision-making. By taking people out of their institutional environments, sitting them down in a room with people from other institutions, and encouraging them to talk openly and honestly about the policy-making environment, policy dialogues shift participants outside of their institutional roles, emphasizing the bigger picture and the multiple perspectives and competing interests involved. This disruptiveness also has the potential to benefit ethical reflection, encouraging reflection not just on the narrowly defined health endpoints of policies, but also their broader social and personal impact.

The need to ‘balance’ and ‘trade off’ values is a feature of value pluralism – the idea that there are at least two values that are not reducible to one another. Values will sometimes be in conflict – choices which promote one value will come at a cost of neglecting another. For example, equity and autonomy are in tension in a policy option that will reduce health inequalities but will also require restrictions on the personal freedoms of some or all citizens; health benefit and privacy will come into conflict when the policy option with the best predicted health outcomes requires intrusive or very broad surveillance or monitoring. Sometimes values will be mutually conducive, when promoting one value also promotes others. Balancing values involves deciding how much to relatively prioritize different values in decision-making and choosing options with an optimal overall mix. The best option might nonetheless involve non-trivial compromises, such as deprioritizing some values in order to prioritize others. Even if policy dialogues only needed to consider the health benefit and equity implications of different policy options, deliberations would likely need to engage in ethical balancing. Including other social values into the mix complicates things further – and moreover, the expected impacts of policies will be evidenced in different ways and there will be different levels of uncertainty around both the size and the nature of effects.

At an abstract level, choosing between different value sets can feel bewildering. Without knowledge of the specific climate in which a decision is being made, and details about the options in question and their effects, there is a limited basis on which to prioritize autonomy over equity, or professional discretion over health benefit, short of insisting on the importance or certain values, or asserting a ranking of values. In the context of a specific decision, however, things may be a little easier. For one thing, policy dialogues do not necessarily involve choices between mutually exclusive options – the best solution to a problem will sometimes be a combination of several policy mechanisms (it is for this reason that we do not focus on ranking alternative options, unlike some ethical frameworks [77]). This can, to some extent, relieve participants from having to choose between options. Moreover, policy options will not necessarily be fixed in their form, so rather than trying to determine whether the costs of some option are worth the benefits, internal balancing might instead consider whether there might be any way of mitigating the costs of an option through changes to the mechanism or use of other concurrent policies. Of course, this will not always be possible – sometimes costs are necessary for policy success.

Perhaps most importantly, balancing takes place within a context of trying to tackle a particular health issue in a given social and cultural context. The appropriateness of options will be constrained by their expected effectiveness at addressing the issue in question. Too great a concern for other considerations will lead to suggestions and recommendations which do not meaningfully address the topic of the policy dialogue, even though they may show the utmost concern for equity, privacy, autonomy and other social values. While population health is not the only consideration in play, the value of options is limited if they are unlikely to in fact make a significant difference to practice, behaviour and, ultimately, outcomes in relation to the issue under consideration. When an issue is a high priority, particularly if action is urgently required to avoid a disastrous outcome, the question for policy dialogue participants to consider might not be whether the costs of taking action are too high, but whether they can afford not to take action. The task in emergency scenarios is not necessarily to choose the ‘most ethical’ action out of all possible actions, but to identify actions that are likely to generate or prevent certain outcomes and that do not also generate excessively bad effects. Introducing an ethical lens to policy dialogues does not involve replacing the pragmatic deliberation about policy options with abstract moral dilemmas but rather ensuring that deliberation includes broader reflection on the implications of policy options and actions.

We have suggested that appropriate inclusion of substantive values in policy dialogues requires genuine deliberation about the broader impact and consequences of policy options, and careful reflection on how important negative consequences are in relation to public health ends. There is no quick fix or standardized value framework which can be invoked to ensure that decisions correctly balance costs and benefits. Good deliberation about values is a part of and an extension of good deliberation in policy dialogues, not a separate activity.

## Conclusions

### Expanding effectiveness as a guiding value for policy dialogues

Policy dialogues are highly pragmatic policy-making tools, which exist because of known obstacles to developing and implementing effective, evidence-informed policies. Effectiveness, both in relation to policy impact and public health outcomes, is therefore central to the design and execution of any policy dialogue. To invoke procedural or substantive values that risk undermining these ends risks rendering policy dialogues ineffective, and leaving their value as policy-making tools unclear. We have argued that a legitimate and good policy dialogue will operate with an expansive conception of effectiveness in two senses. First, it will recognize that policy impact does not just mean generating discrete policies, but also influencing the way that policy-makers and policy-making processes operate in more subtle ways. Secondly, it will recognize that public health outcomes must be understood in the context of the broader flourishing of people and communities and that unreflective pursuit of narrowly defined health outcomes can have indefensible consequences. Broadly, legitimate and good policy dialogues will operate with a nuanced and critical understanding of what ‘success’ means, both in relation to whether policy change has been achieved, and in relation to what policy change achieves.

To help those designing and executing policy dialogues to engage in ethical thinking, we have identified some guiding questions (Table 1). These are not intended to be a checklist or recipe for good policy dialogues, but rather to stimulate ethical reflection on the aims and values that are reflected in the design of policy dialogues and in deliberations, including the core characteristics we identified as procedural values for policy dialogues and many of the substantive values discussed in the Deliberating about values in practice section. There is no right answer to any of these questions, nor will answers necessarily indicate an obvious solution to difficult policy problems – rather they aim to enrich discussion and ensure that deliberation incorporates important ethical consideration.

**Table 1:** Guiding questions for policy dialogues

In designing legitimate policy dialogues, organizers should consider:		
1	Core characteristics	• Is the policy dialogue focused on a high-priority issue, evidence-informed, action-oriented, participatory and deliberative?
2	Objectives	• How should these core characteristics be elaborated and specified in this context, to ensure that the policy dialogue has meaningful policy and public health impact?
3	Contributions	• What is needed from organizers, researchers and donors to ensure that the policy dialogue has these characteristics and meets these objectives? What is needed from participants?
In thinking about what matters, facilitators and participants should consider:		
4	Aims	• What is the aim of policy-making in this area? • What must policies achieve to address the public health issue effectively?
5	Intended impact	• What are the intended and expected health and social outcomes of the policy options under consideration? For example: • What benefits will be attained? • What harms or problems will be prevented? • How certain it is that policy options will achieve these intended outcomes?
6	Broader impact	• Aside from the intended and expected health outcomes, what is the likely impact of the policy options under consideration? For example: • What costs and benefits are not captured in the evidence? • What costs and benefits are difficult or impossible to measure? • What might the broader societal or institutional consequences of this policy be? • How might things go wrong?
7	Who is impacted	• Who will be impacted by the policy and in what ways? For example: • Who will benefit, who will not benefit and on whom will the costs fall? • Will the same people who benefit also endure costs? • Will people who are already disadvantaged be further disadvantaged? • Whose perspectives and what sets of purposes, inform the identification and framing of the problem and the proposed solutions? Has anyone been left out of discussions?
In evaluating policy options, facilitators and participants should consider:		
8	Non-negotiables	• Are there any outcomes that absolutely must be avoided or must be achieved if the policy is to count as successful?
9	Minimizing costs	• How can the policy options be combined or reconceived to reduce the social costs and negative impact? • Are compromise solutions in fact likely to achieve the aims of the policy and address the public health issue in question effectively?
10	Perspectives	• How do the proposed policy options, and their balance of benefits and burdens, look from the perspective of your different roles in life – not just in relation to your job or institutional affiliation but as a parent, a carer, a patient and user of public services, a citizen, a human, and so on?

We cannot offer a simple schema or list of values for ensuring legitimate and good policy dialogues; there is no shortcut to meaningful ethical deliberation. Policy dialogues require a kind of context sensitivity and openness to stakeholder input which precludes any prior exhaustive statement of relevant values and how to balance them – indeed, the identification and definition of values and consideration of trade-offs between them is a crucial part of the policy dialogue process itself. This openness is necessary primarily because, for them to serve their knowledge translation purposes, policy dialogues have to be extremely pragmatic and responsive to particular social and political realities. The questions in Table 1 therefore seek to stimulate deliberation on crucial ethical matters without over-prescribing what participants can bring to the table.

In some respects, our message is simple: those developing and participating in policy dialogues need to themselves engage in ethical reasoning. This does not mean they have to be trained philosophers or learn moral theory, but it does entail adopting a critical and reflective attitude towards their aims and values and the aims of the policy dialogue. Because of this, there is not an established, exhaustive framework which can be applied from the outside, it has to be built from within. And, moreover, it has to be rebuilt in each policy dialogue. While this might seem to be demanding, it also highlights that ethical thinking is not a separate activity but a part of and an extension of ordinary problem solving and decision-making. Good policy dialogues already engage in such ethical thinking insofar as they engage in meaningful, expansive deliberation about the value and limits of public health policies and policy-making.

### Limitations

We have tried to keep the function of policy dialogues in view throughout this paper, and our ethical analysis of policy dialogues has, for the most part, taken them on their own terms. This represents a notable limitation of our project – we have not offered a substantial critique of the aims of policy dialogues, nor how they endeavour to secure them. We have broadly assumed that efforts to support and promote the use of evidence in policy-making processes, and the use of structured, evidence-informed deliberations to do so, are worthwhile and valuable. A broader ethical analysis might also explore how policy dialogues depend on and interact with existing institutions and networks. Policy dialogues are positioned within existing decision-making processes and do not seek to supersede or replace them; as such, even in democratic societies, institutional factors, interest groups and dominant ideas and values will often have much more power in shaping the policy-making process than a dialogue itself.

We have taken as our starting point the fact that policy dialogues are used, and increasingly so, across the world in policy-making. Recognizing that their quality and enactment is variable, we have therefore chosen instead to offer some reflections on how policy dialogues might attend more explicitly and closely to their ethical implications and content, with a view to shaping current practice. In adopting this pragmatic ethical approach, our work is aligned with emerging bioethical scholarship which emphasizes the valuable contribution of philosophical analysis that starts from a relatively detailed and realistic account of actual practice and attempts to address problems and questions that emerge from it [76, 87–89]. Rather than prescribing rules or criteria to which policy-makers and practitioners must conform if they are to reach some standard of ‘ethical’ practice, philosophers working in this vein seek to build ethical thinking into the practices themselves, by encouraging a greater degree of reflectiveness on their implications and limitations. Nonetheless, if future research calls the role of policy dialogues as knowledge translation tools into question, there would be call for a more searching critique of these practices.

## Data Availability

Data sharing is not applicable to this article as no datasets were generated or analysed during the current study.

## Annex: Formal search strategy and exclusions

The search strategy detailed here (Table 2) sought to identify literature that appraised deliberative dialogue as a policy-making tool, with a view to understanding how ethical values are currently considered in this context – recognizing that there are a number of different names for the deliberative tools used in policy-making. The inclusion of ‘policy’ as a keyword aimed to limit literature to the policy context. We settled on a relatively inclusive scope because we were interested in the extent to which ethical considerations are not discussed in relation to policy dialogues, as well as the ways in which they are discussed. This was not a systematic literature review, and it sought to generate a rich and realistic understanding of policy dialogues and the context in which they operate, rather than an exhaustive appraisal of the approach or the field.

**Table 2:** Formal search strategy

Database name	Subsidiary databases/indexes	Search term	Other search limits
Ovid	Including the following databases: • Embase <1974 to present> • Ovid MEDLINE(R) and Epub Ahead of Print, In-Process, In-Data-Review & Other Non-Indexed Citations and Daily <1946 to present> • Global Health <1973 to present> • APA PsycInfo <1806 to present> • Social Policy and Practice <202101>	(policy).mp. AND ("policy dialogue*" OR "deliberative dialogue*" OR "stakeholder deliberation" OR "deliberative engagement" OR "public deliberation").m_titl	• English language • Deduplicate
Web of Science Core Collection	1900–present. Including the following indexes: • Science Citation Index Expanded (SCIE) • Social Sciences Citation Index (SSCI) • Arts & Humanities Citation Index (AHCI) • Conference Proceedings Citation Index-Science (CPCI-S) • Conference Proceedings Citation Index–Social Science & Humanities (CPCI–SSH) • Emerging Sources Citation Index (ESCI)	TI=(("policy dialogue*" OR "deliberative dialogue*" OR "stakeholder deliberation" OR "deliberative engagement" OR "public deliberation")) AND TS=(policy)	• English language • Exclude: meeting abstracts; proceedings papers; book reviews; reviews; news items
ProQuest Political Science	1985–present	noft(policy) AND ti(("policy dialogue" OR "policy dialogues") OR ("deliberative dialogue" OR "deliberative dialogues") OR "stakeholder deliberation" OR "deliberative engagement" OR "public deliberation")	• English language • Exclude: blogs, podcasts and websites; conference papers and proceedings; magazines; newspapers; wire feeds; trade journals; book reviews

After removing duplicates, as well as Wire Feeds, newspaper and magazine articles, book reviews and conference abstracts, PM reviewed the titles and abstracts of all references captured by the searches. Items were excluded if they (a) concerned non-dialogic stakeholder engagement, (b) concerned public engagement rather than broader stakeholder engagement or (c) did not appraise policy dialogues or deliberative dialogues as a method. Papers which took a specific policy dialogue as their subject were included if they also offered some degree of analysis of the method itself. After retrieving the full text of all remaining articles, PM read them to make a final assessment as to their relevance, guided in her assessment by the question: ‘does this paper provide insights into the ethical landscape of policy dialogues?’

The initial search of seven databases generated 283 results. After removing 86 duplicates and four book reviews/conference abstracts, the titles and abstracts of the remaining 193 results were reviewed to exclude those not meeting inclusion criteria. Of the 61 results designated for full text review, one paper was inaccessible. A further five papers were excluded following full text review. Notes were taken on the remaining 55 papers, which were then subject to thematic analysis.

## Abbreviations

A4R: Accountability for reasonableness
EVIPNet: Evidence-Informed Policy Network
HFSS: High in fat, salt and sugar
HTA: Health technology appraisal
